# Complete Genome Sequence of Proteus mirabilis Siphophage Saba

**DOI:** 10.1128/MRA.01094-19

**Published:** 2019-10-10

**Authors:** James Nguyen, Laith Harb, Russell Moreland, Mei Liu, Jason J. Gill, Jolene Ramsey

**Affiliations:** a Center for Phage Technology, Texas A&M University, College Station, Texas, USA; Queens College

## Abstract

Proteus mirabilis is a Gram-negative enteric bacterium associated with complicated human urinary tract infections. Here, we present the complete genome annotation for P. mirabilis siphophage Saba. With a 60,056-bp genome and 75 predicted genes, Saba is most similar at the nucleotide and protein levels to phage Chi and Chi-like viruses.

## ANNOUNCEMENT

Proteus mirabilis is a Gram-negative bacterium commonly found in soil, stagnant water, and sewage and is associated with various animal gastrointestinal tracts (1). P. mirabilis, known for its highly motile swarming lifestyle and formation of biofilms on catheters, is a leading cause of complicated urinary tract infections in humans (2). Bacteriophage applications to mitigate P. mirabilis disease are being explored (3), which led us to isolate and annotate *P. mirabilis* phage Saba.

The source for Saba was filtered (0.2-μm-pore-size filter) wastewater in College Station, TX. Host P. mirabilis strain ATCC 29906 was cultured aerobically at 37°C in nutrient broth/agar (BD). Phages were isolated by the soft-agar overlay method of Adams (4), and small phages were selected by the ability to pass through an Amicon Ultra-15 spin filter with a nominal 30-kDa molecular weight limit (Millipore-Sigma). To determine phage morphology, samples were negatively stained with 2% (wt/vol) uranyl acetate and imaged by transmission electron microscopy at the Texas A&M Microscopy and Imaging Center (5). Saba genomic DNA was purified according to Summer’s modified shotgun library protocol (6). The genome was prepared as Illumina TruSeq libraries with the Nano low-throughput kit and sequenced on an Illumina MiSeq platform using V2 500-cycle chemistry for 250-bp paired-end reads. The 1,421,963 total reads in the phage-containing index were quality controlled using FastQC (http://www.bioinformatics.babraham.ac.uk/projects/fastqc/). FASTX-Toolkit v0.0.14 (http://hannonlab.cshl.edu/fastx_toolkit/) was used for read trimming, and then assembly into a single contig at 44.6-fold coverage was accomplished with SPAdes v3.5.0, with default parameters (7). Contig completion was confirmed by PCR off the ends (forward primer, 5′-TTTACCTTGGAGCACTTGATCG-3′, and reverse primer, 5′-GTAGGGCGGTATTGCGTTTAT-3′) and Sanger sequencing. PhageTerm was used to predict the genomic terminus type, but Saba was unclassified by this method (8). Genes were predicted from Glimmer v3.0 and MetaGeneAnnotator v1.0 outputs (9, 10). Potential tRNA genes were detected with ARAGORN v2.36 (11). Putative Rho-independent terminators were assessed with TransTermHP v2.09 (12). Protein functions were predicted primarily with InterProScan v5.33-72 and BLAST v2.2.31 searched with a 0.001 maximum expectation value cutoff against the NCBI nonredundant and UniProtKB Swiss-Prot/TrEMBL databases (13–15). Putative transmembrane domains were analyzed with TMHMM v2.0 (16). Whole-genomic DNA sequence similarity with top-related phage was calculated using the progressiveMauve v2.4.0 algorithm (17). The tools mentioned here (run at default parameters unless otherwise stated) are all accessible in the Galaxy instance hosted by the Center for Phage Technology at https://cpt.tamu.edu/galaxy-pub (18), and annotation was performed in the Apollo instance (19).

The 60,056-bp Saba siphophage genome has 48.8% G+C content. With 75 coding sequences predicted, Saba has 93.5% coding density. Within the nonredundant database, Saba is most similar to *Proteus* phage pPM_01 (50.94% nucleotide identity and 55 shared proteins, GenBank accession number KP063118) and *Proteus* phage PM87 (50.83% nucleotide identity and 48 shared proteins, GenBank accession number MG030346), as well as enterobacterial phage Chi (29.45% nucleotide identity and 40 proteins, GenBank accession number JX094499). Given these similarities to Chi and Chi-like viruses, Saba may also be flagellotropic.

### Data availability.

The genome sequence and associated data for phage Saba were deposited under GenBank accession number MN062188, BioProject number PRJNA222858, SRA run number SRR8892147, and BioSample number SAMN11408687.
